# Vitamin B12 Deficiency Mimicking Thrombotic Thrombocytopenic Purpura: A Case Report and Review of Literature

**DOI:** 10.7759/cureus.63478

**Published:** 2024-06-29

**Authors:** Bayle Smith-Salzberg, Sylvester Homsy, Burak Erdinc, Mohan Preet

**Affiliations:** 1 Hematology and Oncology, State University of New York Downstate Health Sciences University, Brooklyn, USA

**Keywords:** vitamin b12 deficiency, schistocyte, thrombocytopenia, hemolytic anemia, acquired ttp

## Abstract

Vitamin B12 (cyanocobalamin) deficiency can lead to ineffective erythropoiesis, intramedullary hemolysis, and, in severe cases, neurologic deficits. Some of those findings are also features of thrombotic microangiopathies, specifically thrombotic thrombocytopenic purpura (TTP), and the distinction between both entities could sometimes be challenging. While the treatment of the former consists of enteral or parenteral repletion, the treatment of TTP is more complex and time-sensitive. For that reason, refining diagnostic strategies is crucial to avoid misdiagnosis and unnecessary interventions. Here is an example of a potential life-threatening hemolysis caused by vitamin B12 deficiency with acute onset neurologic symptoms, which resolved with B12 repletion.

## Introduction

Vitamin B12 is stored in the liver for three to four years making nutritional vitamin B12 deficiency rare in individuals with a regular diet and no underlying conditions involving their small bowel. However, deficiency can arise through various mechanisms, including pernicious anemia or atrophic gastritis where antibodies against parietal cells and intrinsic factors prevent B12 absorption. Additionally, bariatric surgery, inflammatory bowel disease, intestinal resections, veganism, alcohol use disorder, and long-term use of certain medications may contribute to vitamin B12 deficiency [1-3]. This entity is often asymptomatic or mild and manifests with symptoms such as fatigue, jaundice, and palpitations [1]. In severe cases, it can progress to paresthesias, peripheral neuropathy, loss of proprioception and vibration sense, and subacute combined degeneration, which may become permanent [2,3]. In around 10% of vitamin B12 deficiency cases, patients present with hemolysis, and around 25% of those exhibit a triad of hemolytic anemia, thrombocytopenia, and schistocytosis [4,5]. These clinical scenarios closely resemble thrombotic microangiopathy (TMA), leading to the designation of pseudo-TMA in such cases. TMAs are life-threatening conditions that include thrombotic thrombocytopenic purpura (TTP), hemolytic uremic syndrome (HUS), and disseminated intravascular coagulation (DIC). Given their significant overlapping features, differentiating between vitamin B12 deficiency and TMA can be challenging. Here, we present a case of a 74-year-old woman who was found to have pancytopenia and hemolysis, initially suspected to be TMA but found to have severe vitamin B12 deficiency. 

## Case presentation

**Figure 1:**
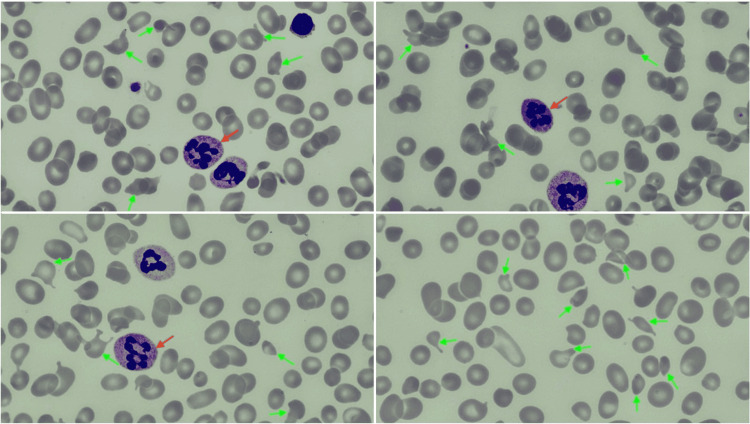
Peripheral smear at presentation showing hyper-segmented neutrophils (red arrows) and schistocytes (green arrows) (100x magnification)

A 74-year-old African-American woman with essential hypertension and rheumatoid arthritis presented to the hospital with acute onset of weakness, fatigue, gait instability, and slurred speech for four days, previously without any neurologic symptoms. On physical examination, vital signs were normal outside of a blood pressure of 145/80. Neurologic examination was consistent with a right nasolabial fold droop, weakness in her left arm and leg, and slurred speech, and hence a stroke code was called; the rest of the physical examination was within normal limits. Brain imaging with magnetic resonance imaging (MRI) brain was negative for any intracranial pathologies. The patient was found to have megaloblastic anemia (hemoglobin of 7 g/dL with mean corpuscular volume (MCV) of 118 fL), thrombocytopenia with platelets of 79,000, and normal white blood cell count. Further lab testing showed nonimmune hemolytic anemia with high lactate dehydrogenase (LDH), undetectable haptoglobin, normal reticulocyte count, and negative direct antiglobulin test. Coagulation studies showed a slightly elevated PT of 16 but activated partial thromboplastin time (aPTT) was normal. Examination of the peripheral smear revealed hyper-segmented neutrophils and an increased number of schistocytes (Figure 1).

Due to the low risk of severe ADAMTS13 deficiency as calculated by a PLASMIC score of 4, plasma exchange was not recommended. Prednisone was initiated in light of possible TMA. Later, it was found that the patient had severe vitamin B12 deficiency (<85 ng/mL) with normal folate and was initiated on a daily 1000 mcg intramuscular (IM) cyanocobalamin for seven days, followed by weekly 1000 mcg IM cyanocobalamin for one month, which resulted in gradual improvement of her hemoglobin, platelets, hemolysis markers, and neurological symptoms. Intrinsic factor antibody was positive, which confirmed the diagnosis of pernicious anemia. The patient was eventually discharged after clinical improvement. Repeat blood work in three months showed a complete resolution of laboratory abnormalities (Table 1).

**Table 1:** Lab parameters at presentation and three months after vitamin B12 repletion MCV: mean corpuscular volume; LDH: lactate dehydrogenase

Lab parameters	At presentation	3 months after B12 treatment	Reference range
Hemoglobin (g/dL)	7	15	12-16
MCV (fL)	118	89	80-100
Platelets	79000	392000	150000-400000
LDH (U/L)	1856	363	140-271
Haptoglobin (mg/dL)	<30	91	44-215
Indirect bilirubin (mg/dL)	2.4	0.1	0.1-0.7
Vitamin B12 (pg/mL)	<86	740	165-1000

## Discussion

This case underscores the challenge of distinguishing between vitamin B12 deficiency and TMA. TTP poses a significant risk of major thrombotic events or death if treatment is delayed, emphasizing the critical need for accurate diagnosis and prompt intervention [6]. 

The pathophysiology of pseudo-TMA in vitamin B12 deficiency is not fully understood, but there are known overlapping features between B12 deficiency and TMA. Vitamin B12 is a cofactor for two enzymatic reactions. First, the reaction that converts L-methylmalonyl-coenzyme A to succinyl-coenzyme A in odd-chain fatty acid metabolism. An increase in methylmalonic acid is, therefore, a sign of vitamin B12 deficiency. Vitamin B12 is also a cofactor for methionine synthase, which converts homocysteine and methyl-tetrahydrofolate to methionine and tetrahydrofolate. This reaction is crucial for regenerating tetrahydrofolate, a cofactor required for the production of nucleic acids for DNA. As a result, patients with a vitamin B12 deficiency have impaired DNA synthesis, which results in macrocytosis in red blood cells (RBCs) and hyper-segmentation in granulocytes [7]. Additionally, the accumulation of homocysteine disrupts glutathione synthase and leads to an increase in reactive oxygen species, which aggravates RBC integrity [4]. Thus, relative homocysteinemia caused by vitamin B12 deficiency can cause intramedullary hemolysis [7,8]. Finally, vitamin B12 has been shown to be necessary for myelination in the central nervous system and its deficiency can cause neurological impairment [9]. 

Similarly, TTP is characterized by a pentad of hemolytic anemia, thrombocytopenia, renal symptoms, neurological symptoms, and fever. In TTP there is a deficiency of ADAMTS13, a metalloprotease crucial for preventing the accumulation of procoagulant forms [10]. Insufficient ADAMTS13 allows large multimers of Von Willebrand factor to accumulate in the microvasculature, forming platelet-containing thrombi. Vessel-bound thrombi shear RBCs, causing microangiopathic hemolytic anemia and characteristic schistocytes on peripheral blood smears. 

Both TTP and vitamin B12 deficiency may manifest with peripheral hemolysis, thrombocytopenia, and neurological symptoms. While TTP can cause kidney dysfunction, one of the gastrointestinal symptoms of B12 deficiency is diarrhea, which would lead to renal impairment [11]. However, their clinical management differs significantly. TTP is life-threatening and it is essential to quickly diagnose and treat this condition with plasma product therapy. In contrast, vitamin B12 deficiency is easily reversible with vitamin B12 supplementation, although neurological symptoms may become irreversible with a delay in diagnosis and treatment [2,3]. 

Given the potential lethality of TTP, plasma product therapy is often initiated before definitive test (ADAMTS13) results confirm the diagnosis. Previous studies have reported cases of vitamin B12 deficiency with pseudo-TMA receiving plasma product therapy, with associated risks and complications [12-14]. For instance, in a meta-analysis by Tun et al., of 36 patients reported with severe B12 deficiency and 38.8% of reported cases received plasma product therapy [12]. In another study of 14 patients who received plasma exchange for vitamin B12 deficiency-related pseudo-TMA, two had major complications from the treatment [13]. There was a 2.3% mortality rate and a 24% risk of major complications due to plasma exchange treatment seen in an Oklahoma TTP registry over 15 years [14]. Thus, it is important to distinguish vitamin B12 deficiency from TTP to avoid treatment with potentially harmful side effects.

Our patient presented with hemolytic anemia, thrombocytopenia, and neurologic symptoms, three of the five pathognomonic TTP symptoms. We, therefore, initially suspected TTP but ultimately considered a nutritional deficiency because of a low PLASMIC score and the onset of neurological symptoms, which usually appear at the latest stages of TTP, without a fever, often appearing first. 

The PLASMIC score is a validated tool that has been shown to correlate well with ADAMTS13 levels [15-17]. This calculator takes into account platelet count, creatinine, MCV, INR, evidence of hemolysis, and prior malignancy or transplant. Our patient had a PLASMIC score of 4 (low risk), and so we were able to avoid unnecessary plasmapheresis therapy. However, it is worth noting that there have been several cases of severe vitamin B12 deficiency scoring a 5 or higher (intermediate risk) on the PLASMIC score, indicating the complexity of diagnosis [1,8,18]. 

While the PLASMIC score proved useful in our case, other factors commonly employed in strengthening the B12 deficiency diagnosis, such as reticulocyte count and LDH level, presented challenges. Studies have shown that the reticulocyte production index can be used to distinguish vitamin B12 deficiency from classical hemolytic anemias since it is notably decreased in vitamin B12 deficiency [13]. However, our patient had normal reticulocyte counts. Additionally, patients with pseudo-TMA associated with vitamin B12 deficiency generally have significantly higher LDH levels, greater than 2500 IU/L, than patients with true TTP [5,11,13]. Our patient is one of the exceptions to this trend with an LDH of 1856 IU/L at presentation. It is also important to note that there are cases of patients with TTP who have LDH levels that exceed 2500 IU/dL, so this factor alone cannot be used to rule out TTP [14]. Finally, our patient’s B12 level of <83 pg/mL provided conclusive evidence for the diagnosis. 

Integrating tools like the PLASMIC score with other key clinical and laboratory findings, such as reticulocyte count and LDH level, may guide accurate diagnosis and treatment decisions, ensuring optimal patient outcomes.

## Conclusions

This case highlights the critical need for accurate differentiation between vitamin B12 deficiency and TMA, as their overlapping symptoms can lead to misdiagnosis and inappropriate treatment. While they may present similarly, TTP poses a life-threatening risk requiring prompt intervention with plasma exchange, and vitamin B12 deficiency is easily reversible with supplementation. This case underscores the importance of utilizing various diagnostic tools and emphasizes the need for further refining strategies to ensure optimal patient outcomes and avoid unnecessary interventions.
